# Pathogen infection drives patterns of nutrient resorption in citrus plants

**DOI:** 10.1038/srep14675

**Published:** 2015-09-30

**Authors:** Jirong Cao, Chunzhen Cheng, Junjie Yang, Qibing Wang

**Affiliations:** 1 State Key Laboratory of Vegetation and Environmental Change, Institute of Botany, Chinese Academy of Sciences, Beijing 100093, China; 2 Institute of Horticultural Biotechnology, Fujian Agriculture and Forestry University, Fuzhou, Fujian 350002, China

## Abstract

Nutrient resorption processes in the plants infected by pathogen remain poorly understood. Huanglongbing (HLB) is a destructive disease of citrus. HLB-pathogen ‘*Candidatus* Liberibacter asiaticus’ grows specifically in the phloem of hosts and may cause problems in the plant vascular system after infection. Therefore, it brings a great concern about the phloem nutrient transport and nutrient intra-cycling in HLB-affected plants. We investigated the effects of ‘*Ca.* L. asiaticus’ infection on nitrogen (N) and phosphorus (P) concentrations and resorption in different citrus species (i.e. *Citrus reticulata*, *Citrus limon* and *Citrus maxima*). HLB-pathogen infection had distinctive impacts on nutrient resorption in different species. P resorption efficiency substantially decreased in infected *C. reticulata* plants relative to the healthy plants in summer, which may account for the marked decrease in the average fruit yield. P resorption was more efficient in infected *C. limon* plants than in the healthy plants. However, for *C. maxima* plants, HLB had no significant effects on N:P ratio in live leaves and resorption efficiency as well as on fruit yield. Keeping efficient internal nutrient cycling can be a strategy of citrus species being tolerant to HLB.

Citrus Huanglongbing (HLB) disease is one of the most devastating diseases of citrus and is threatening citrus industry worldwide^1^,^2^. HLB-associated pathogens are the phloem-limited, Gram-negative bacteria named ‘*Candidatus* Liberibacter spp.’, including the three species of ‘*Ca*. L. asiaticus’, ‘*Ca*. L. americanus’ and ‘*Ca*. L. africanus’^3^. These bacteria are transmitted chiefly by psyllids *Diaphorina citri* in Asia and America and by *Trioza erytreae* in Africa^4^. Timely monitoring of citrus trees for HLB symptoms is critical for early detection and management of the disease, but there are no effective measures to control HLB now. Removal of infected trees and treatments with insecticide against psyllids are the most common ways to control the spread of HLB pathogens locally^1^,^5^.

One of the most distinct symptoms of HLB is the development of yellow shoots on infected trees^6^. HLB also induces other characteristic symptoms, such as blotchy mottle leaves and lopsided fruits with color inversion and aborted seeds^7^. However, it is difficult to diagnose HLB by symptoms. Some HLB symptoms can be masked by the symptoms of other diseases. In particular, the leaf symptom of ‘*Ca*. L. asiaticus’-infected plants is easy to be confused with the symptom caused by nutrient deficiency, especially in an early infected stage^8^. Therefore, much of research has attempted to find out the relations of nutrients and HLB symptom expression^9^,^10^,^11^,^12^.

Nutrients are important factors in disease control, because nutrients affect plant, pathogen and microbial growth and their interactions. Dordas^13^ reviewed literatures on the relationships between mineral nutrients and plant disease and concluded that most of essential nutrients influenced the severity of plant disease. It seems plausible that nutrient supplements could reduce the severity of disease symptom, yet there are many findings on both sides of this debate^13^,^14^. For instance, the severity of crown and root rot of tomato was reduced by supplements of nitrate-nitrogen and copper, but increased by ammonium-nitrogen, zinc and manganese^15^. Likewise, for HLB, some studies showed that the application of phosphorus (P) to infected citrus trees improved plant growth and yield^10^,^16^. However, Gottwald, Graham^12^ revealed that the nutritional supplements (P and micronutrients) had no effects on growth, fruit quality and yield of ‘*Ca*. L. asiaticus’-infected trees. Furthermore, Zambrosi *et al.*^17^ found that the great proportion of P in the flowers and fruits were from the remobilized P in citrus plants, and suggested that P resorption might be more important than P uptake to meet the demands of new vegetative and reproductive growth of citrus plants, even with adequate nutrient supply. Therefore, P resorption could play a vital role in the interaction between citrus plant and HLB-pathogen. However, little is known about the nutrient resorption in the plants infected by HLB pathogen. Nutrient resorption is a key strategy for conserving nutrients in plants. In this process, plants remove nutrients from senescing tissues and transport them through the phloem to other living tissues^18^,^19^,^20^. Phosphorus is readily phloem-mobile in plants^21^. It has been reported that callose deposition in phloem sieve tubes can restrict phloem transport in citrus plants infected with ‘*Ca.* L. asiaticus’^22^,^23^. Moreover, it was reported that ‘*Ca.* L. asiaticus’ infection reduced P concentration by 35% in ‘*Ca.* L. asiaticus’-infected citrus plants compared with the healthy plants^10^. In this context, we hypothesized that (1) P would be a limiting nutrient in the citrus plants infected with ‘*Ca.* L. asiaticus’, and (2) the HLB-pathogen infection would cause the change in P resorption in the host plant.

Few studies have focused on the responses of different citrus species to HLB to date. Besides, the effects of HLB on nitrogen (N) in the host plants are rarely reported^11^. In this study, we investigated the effects of ‘*Ca*. L. asiaticus’ infection on the nutrient resorption in citrus plants among different species. We were interested in understanding how N and P concentrations in leaves changed responding to ‘*Ca*. L. asiaticus’ infection, how the infection affected N and P resorption in citrus plants, and whether there were convergent responses of nutrient resorption to HLB among different citrus species.

## Results

### Nutrients in live and senesced leaves

**Figure 1:**
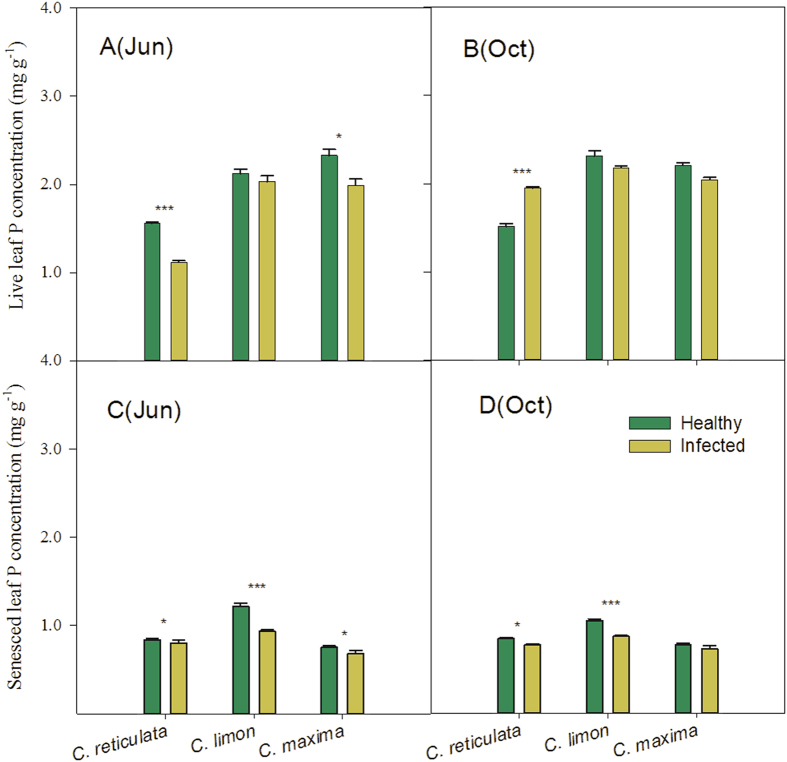
P concentrations in live- and senesced-leaves of different citrus species. Values are mean± Se, n=5. *, ** and *** above bars indicate significant differences at *P* < 0.05, 0.01 and 0.001, respectively, derived from the results of paired *t-*tests.

**Figure 2:**
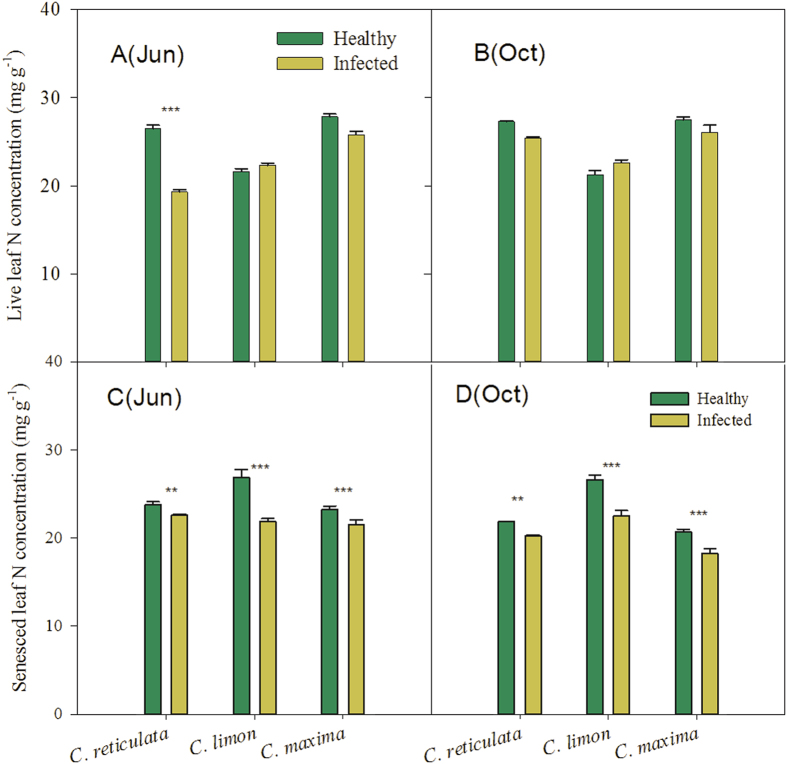
N concentrations in live- and senesced-leaves of different citrus species. Values are mean ± Se, n = 5. *, ** and *** above bars indicate significant differences at *P* < 0.05, 0.01 and 0.001, respectively, derived from the results of paired *t-*tests.

The ‘*Ca*. L. asiaticus’ infection had significant effects on N and P concentrations in live leaves of citrus plants in our study. Even so, most of the N and P concentrations in live leaves of the ‘*Ca*. L. asiaticus’-infected plants still remained at optimum or high levels, according to the criteria shown in Table 1. There were two exceptions. Leaf N and P concentrations in infected *C. reticulata* plants in June dropped to the deficient and low levels. N concentrations in *C. limon* plants, which were deficient in the healthy trees as an unusual case, reached to the low level in the infected trees. Repeated measure-ANOVA based on mixed models shows that different species and sampling dates also have significant effects on concentrations of N and P in live leaves, and that there are interactive effects of species and health status on live-leaf N and P concentrations (*P* < 0.0001; Table 2). For example, in June, the concentrations of live-leaf N and P in *C. reticulata* plants significantly decreased in response to the HLB-pathogen infection, whereas those in *C. limon* plants remained unchanged (Figs 1a and 2a). In October, the P concentration of live leaves in infected *C. reticulata* plants recorded a marked increase (*P* < 0.001), while concentrations of live-leaf N and P in infected *C. limon* plants showed no significant changes, compared with those in the healthy plants (Figs 1b and 2b).

Concentrations of N and P in senesced leaves declined in infected *C. reticulata, C. limon* and *C.maxima* plants, compared with those in the healthy plants (Figs 1c,d and 2c,d). The effects of species and the interactive effects of species and health status on concentrations of N and P in senesced leaves were also significant (*P* < 0.0001; Table 2). In addition, there were no interactive effects of species, health status and sampling dates on N and P concentrations in senesced leaves (Table 2).

### Nutrient resorption efficiency

**Figure 3:**
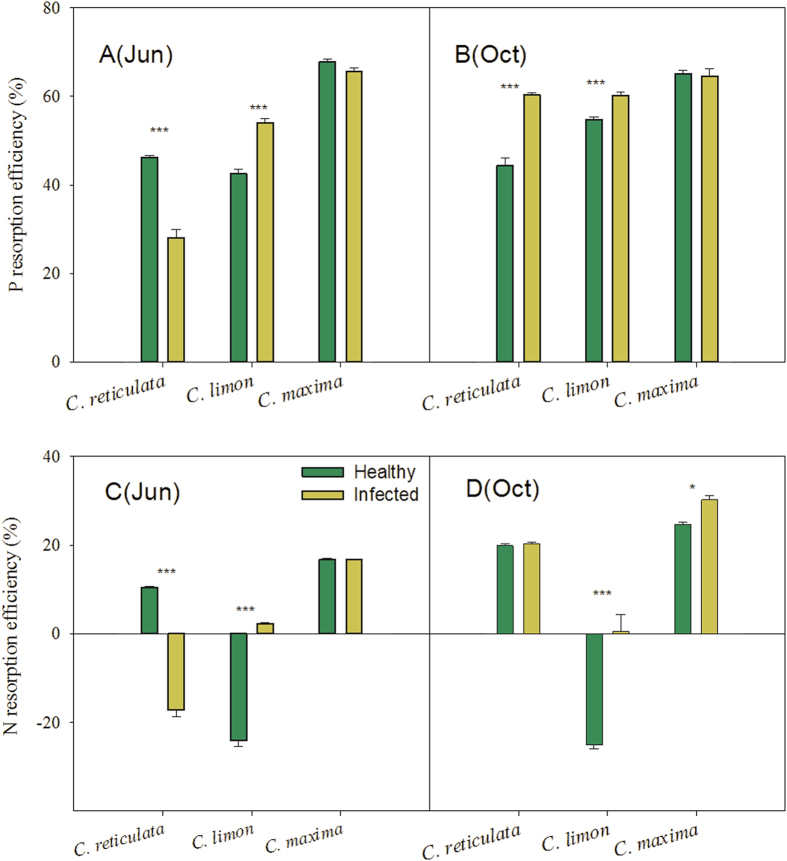
P (a,b) and N (c,d) resorption efficiencies of different citrus species. Values are mean±Se, n = 5. *, ** and *** above bars indicate significant differences at *P* < 0.05, 0.01 and 0.001, respectively, derived from the results of paired *t-*tests.

P resorption efficiency was significantly greater than N resorption efficiency across the three citrus species, regardless of ‘*Ca*. L. asiaticus’ infection (matched pairs analysis *P* < 0.0001; Fig. 3). The HLB-pathogen infection had marked impacts on P resorption efficiencies among different citrus species (*P* = 0.001 for HLB effects, *P* < 0.0001 for species effects, *P* < 0.0001 for their interactive effects; Table 2). P resorption efficiency recorded an overall increase in ‘*Ca*. L. asiaticus’-infected citrus plants compared with healthy plants (*P* < 0.001). Phosphorus resorption was more efficient in infected *C. limon* plants than in the healthy plants (*P* < 0.001; Fig. 3a,b). The P resorption efficiency in *C. maxima* plants showed no significant changes in response to ‘*Ca*. L. asiaticus’ infection in summer and autumn. The fruit production of *C. maxima* plants was not affected by HLB (209.7 kg/tree in the healthy trees and 202.8 kg/tree in the infected trees). However, there were distinct seasonal patterns in P resorption efficiency in infected *C. reticulata* plants. Compared with the healthy plants, P resorption efficiency in HLB-positive *C. reticulata* plants substantially decreased in June, while it increased in October (*P* < 0.001; Fig. 3a,b). N resorption efficiency was also significantly affected by the HLB-pathogen infection (*P* < 0.0001; Table 2) and presented similar patterns to P resorption efficiency (Fig. 3c,d). Especially, N in infected *C. reticulata* plants showed accumulation in the senesced leaves in June. The average *C. reticulata* fruit yield dropped from 21.7 kg/tree in the healthy plants to 4.5 kg/tree in the infected plants.

### Correlations of nutrient concentration and resorption efficiency

P concentrations in live leaves were positively correlated with P resorption efficiencies (*r*^2^ = 0.44 for healthy plants and *r*^2^ = 0.81 for ‘*Ca*. L. asiaticus’-infected plants, *P* < 0.0001; Table 3). Differently, the negative correlation between P concentration in senesced leaves and P efficiency was found in healthy plants (*P* < 0.0001); however, this correlation was not significant in infected plants (*P* = 0.137). N concentrations in live leaves were also significantly correlated with N resorption efficiencies. Significant correlations between N concentrations in senesced leaves and N resorption efficiencies were observed in both healthy and infected plants.

**Figure 4:**
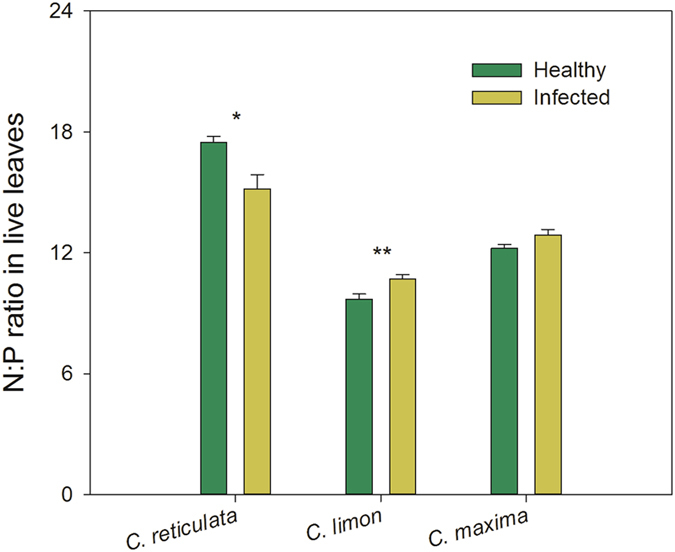
N:P ratios in live leaves of different citrus species. There were no significant differences in N:P ratio between sampling dates for all species, therefore the N:P ratios in June and October were compiled to calculate means ± Se (n = 10) for each species. * and ** above bars indicate significant differences at *P* < 0.05 and 0.01, respectively, derived from the results of paired *t-*tests.

Resorption efficiencies of N and P were significantly correlated with one another (*r* = 0.40, *P* = 0.027 for healthy plants and *r* = 0.83, *P* < 0.0001 for infected plants; Table 3); so were concentrations of N and P in senesced leaves (*r* = 0.75, *P* < 0.0001 for the healthy and *r* = 0.44, *P* = 0.015 fo*r* the infected). A significant correlation between N and P concentrations in live leaves was detected in infected plants (*r* = 0.66, *P* < 0.0001), but was not in healthy plants (*r* = −0.31, *P* = 0.091). There were opposing effects of ‘*Ca*. L. asiaticus’ infection on N:P ratios in live leaves, with a decrease in *C. reticulata* plants and an increase in *C. limon* plants (*P* = 0.03 and *P* = 0.01, respectively; Fig. 4). However, the effects ofthe infection on N:P ratios in senesced leaves were not significant (Table 2). The N:P ratio in live leaves was significantly correlated with P resorption efficiency (*r* = −0.36, *P* = 0.049 for healthy plants and *r* = −0.73, *P* < 0.0001 for infected plants). The correlation of N:P in senesced leaves and P resorption efficiency was significant only in healthy plants (Table 3).

## Discussion

HLB is a highly destructive citrus disease associated with the phloem-limited fastidious ‘*Ca.* Liberibacter spp.’, bringing an unprecedented challenge to citrus fruit production throughout the world. There is no effective method available to control HLB at present. Therefore, it is urgent to understand the interaction of citrus and ‘*Ca.* L. asiaticus’ to unravel the pathogenic processes and develop innovative strategies of disease control. To our knowledge, the study presented here is the first analysis of nutrient resorption in host plants responding to ‘*Ca.* L. asiaticus’ infection.

### Would citrus plants be P limited under HLB-pathogen infection?

A study associated with HLB found a substantial reduction of leaf P in ‘*Ca*. L. asiaticus’-positive citrus plants compared with healthy plants (*C. sinensis*)^10^. Accordingly, we hypothesized that P would be a limiting nutrient in ‘*Ca*. L. asiaticus’-infected citrus species (i.e. *C. reticulata*, *C. limon* and *C. maxima*). Our results were not completely in agreement with this hypothesis. For species *C. limon* and *C. maxima*, P concentrations in live leaves remain at optimum or even high levels in the plants infected with the HLB pathogen (Table 1). However, *C. reticulata* plants were P limited under the HLB-pathogen infection in summer. Mann *et al.*^24^ reported that P was deficient in ‘*Ca*. L. asiaticus’-infected citrus plants (*C. sinensis*). Our data also show that species, health status and their interaction have significant effects on the P in live leaves (*P* < 0.0001; Table 2). Therefore, the hypothesis was not suitable for all citrus species, at least not for *C. limon* and *C. maxima* in the present study. The differences in the responses of different citrus species to HLB, for instance, being sensitive, tolerant or resistant, might be the main reason for the inconsistency of the hypothesis and the results. Meanwhile, other factors such as seasons should be taken into account as well. The results from Folimonova *et al.*^25^ indicate that different species show their tolerance to HLB differently, and generally support our observation on the tolerance of the three citrus species. Our results suggest that the maintenance of optimum or higher P concentrations within the citrus plants can be a defensive strategy against the HLB-bacterial infection.

### Would HLB-pathogen infection cause the change in P resorption in the host plant?

The HLB pathogen inhabits in a specialized niche, that is, the phloem sieve tubes, in host plants^6^. Electron microscope evidence illustrated the deposition of callose in the sieve tubes in citrus plants infected with ‘*Ca*. L. asiaticus’^23^,^26^. The synthesis of pathogen-induced callose is a defense response of the plant immunity that is controlled by signaling pathways^27^. However, the excessive formation of callose in phloem sieve tubes can lead to the restriction of phloem transport in ‘*Ca*. L. asiaticus’-infected citrus plants (e.g. *C. reticulata*, *C. sinensis* and *C. paradisi*)^22^,^23^,^28^. Built upon these previous findings, a focus in our study was the effects of ‘*Ca*. L. asiaticus’ infection on the nutrient resorption through phloem transport in citrus plants. Zhao, Sun^10^ discovered that ‘*Ca.* L. asiaticus’ infection in citrus plants induced miR399, a miRNA highly related to P starvation. Thus, we hypothesized that HLB-pathogen infection would cause the change in P resorption efficiency. Our results show that ‘*Ca*. L. asiaticus’ infection has a marked impact on P resorption efficiency, with an overall increase in infected plants relative to healthy plants. Nutrient resorption is the process in which plants reallocate phloem-mobile nutrients from senescing tissues to storage sites^19^,^29^. Efficiency, as a measure of resorption, is uniquely featured by creating a linkage between nutrient demand (live-leaf nutrient concentration) and nutrient withdrawal (senesced-leaf nutrient concentration)^30^. Intuitively, the putative restriction in the phloem induced by ‘*Ca*. L. asiaticus’ infection would hinder the resorption process, thus making the resorption less efficient. However, the interaction between plant immune system and pathogen is very complex^31^, as was shown in our study.

In the study, we observed a significant negative correlation between P concentration of senesced leaf and P resorption efficiency in healthy plants (Table 3). This result indicates that the trend of P resorption proficiency (lower nutrient concentration in senesced leaves indicates higher nutrient proficiency) is consistent with that of P resorption efficiency in healthy plants. However, this linkage disappeared in ‘*Ca*. L. asiaticus’-infected plants (Table 3). It suggests that under ‘*Ca*. L. asiaticus’ infection, the level to which infected plants reduced P in senesced leaves (measured as proficiency) can not predict the efficiency of reallocating P from senesced leaves to live leaves through phloem transport. Therefore, taking the complementary nature of resorption efficiency and proficiency into consideration as suggested by Killingbeck^29^, we would possibly find a clue to know whether the P phloem transport was normal under the HLB-pathogen infection. The infection had distinctive impacts on the P resorption in the different species. The most remarkable was the pattern in *C. reticulata*. The P resorption efficiency decreased, whereas the P proficiency increased, in the infected *C. reticulata* plants compared with the healthy plants in summer. It turned out that the changes in resorption efficiency and proficiency were contradictory. The increase in P resorption proficiency in infected *C. reticulata* plants indicated that the removal of P from the senesced leaves actually happened. Coupled with the significant decrease in P concentration of live leaf in infected *C. reticulata* plants, it might be a cue revealing that P transport in phloem was abnormal and consequently the P withdrawn from the senesced leaves did not relocate to the live leaves. In fact, the process of nutrient resorption can be divided into two steps, that is, the removal of nutrients from senescing tissues and the transport of these nutrients to storage tissues in perennial plants^29^. Therefore, both steps are equally important. The induction of genes involved in plant defense against the HLB pathogen in *C. reticulata* plants indicates that *C. reticulata* is not completely vulnerable to the infection, though this species is considered to be susceptible to ‘*Ca*. L. asiaticus’ infection by Albrecht and Bowman^32^. From physiological aspect, the results in our study agree on this count. In autumn, a substantial increase in the P resorption efficiency was observed in infected *C. reticulata* plants, relative to the healthy plants. These results to some extent can attribute to the favorable climate for better growth of plants. Our data show that season and the interaction of season and health status have significant effects on P concentration and resorption efficiency (Table 2). After the early summer (the first sampling), the increases of temperature and rainfall can be beneficial for the infected *C. reticulata* plants to overcome the temporary disorder in the P transport and to return to a greater P resorption^33^,^34^,^35^. The P resorption process in the infected *C. reticulata* plants reflected the dynamic interaction between P and the plant-pathogen system. Future study to elucidate the molecular mechanism underlying the HLB-affected phloem transport of P resorption is warranted.

Distinguishing from the pattern in *C. reticulata* plants, the P resorption efficiency in *C. maxima* plants was not affected by the HLB-pathogen infection. The resorption efficiency in infected *C. limon* plants were greater than that in the healthy plants in both seasons. These results suggest that P resorption may be an adaptive trait with great flexibility in some citrus species under the disease stress. Nutrients can affect disease susceptibility, tolerance and resistance in plants^14^. Disease resistance is the ability of a host plant to restrict or suppress the development and reproduction of the pathogen^36^. The tolerance of a host plant is regarded as the ability to maintain its growth and yield under pathogen infection^13^. The transcriptional profiling proves that many genes are differentially regulated in citrus following ‘*Ca*. L. asiaticus’ infection^31^,^37^, demonstrating that the host plant initiates its defense system to cope with the detrimental effects of the pathogen, instead of being passive in the plant-pathogen interaction. However, none of these genes are substantially expressed to suppress the pathogen development during the early stage of the infection^37^. To the best knowledge of authors, complete HLB resistance in citrus species has not been identified yet. However, there are some citrus species considered to be tolerant to HLB^25^,^38^. Disease tolerance is genetically controlled, and thus different species present variations in the tolerance to disease^39^. The results on *C. maxima* and *C. limon* provide a hint that some citrus species could balance tissue nutrient levels by regulating nutrient resorption to improve the tolerance of plant itself to HLB.

### The significance of remobilized nutrients for fruit product

It has been shown that N and P can restrict the pathological effects induced by pathogen infection^13^. The changes in nutrient resorption efficiency will make impacts on other processes such as shoot growth and reproductive output, since nutrient resorption is one of the most important strategies employed by plants to conserve nutrients^18^. The results in our study imply that more efficient resorption of N and P may promote the growth of infected plants to counteract the destructive effects of HLB-pathogen infection. The process of nutrient resorption conserves nutrients in plants and reduces the dependence of plants on source in the soil^29^. The relative importance of nutrient uptake and resorption for growth varies in different plants. For citrus plants, P remobilization may be dominant over P uptake to meet the demands of new vegetative and fruit growth^17^. There is no doubt that fruit productivity is a major concern for citrus growers upon ‘*Ca*. L. asiaticus’-infection of citrus plants. The production of fruits requires prominent reallocation of nutrients from senescing leaves to reproductive tissues^40^, because fruits contain greater N and P concentrations than vegetative tissues^41^. Particularly, the remobilized P in citrus plants accounts for 72–97% of the P in flowers and fruits, even when P supply is sufficient^17^. In our study, the HLB-pathogen infection affected P resorption with important feedback on the plant productivity. The substantial decrease in P resorption efficiency in infected *C. reticulata* plants relative to the healthy plants in summer, may account for the marked decrease in the average fruit yield in autumn (21.7 kg/tree vs. 4.5 kg/tree in the healthy vs. the infected trees, respectively). However, for *C. maxima* plants, in which the HLB-pathogen infection did not significantly change the resorption efficiency, the fruit yield was not affected by HLB. These results can be understood as a strategy of species *C. maxima* being tolerant to HLB, i.e. keeping efficient internal nutrient cycling to sustain the fruit growth of the infected plants.

### Effects of other factors on nutrient resorption

As discussed above, HLB-pathogen infection significantly drove the patterns in nutrient resorption in citrus plants. The effects in species and interactions of species and health status contributed to these patterns as well. In addition, live-leaf N:P ratio was significantly correlated with P resorption efficiency, accounting for 53% of the variance in P resorption efficiency in ‘*Ca*. L. asiaticus’-infected plants. Importantly, the balanced nutrition in plants can facilitate the expression of disease resistance or tolerance^14^. Güsewell^42^ reported that high N:P ratios could promote the tolerance of plant species under stress. In our study, the decreases in both live-leaf N:P and fruit yield in infected *C. reticulata* plants relative to the healthy plants indicate that species *C. reticulata* is sensitive to the HLB-pathogen infection. However, species *C. maxima* showed the tolerance to HLB, since HLB did not make an impact on live-leaf N:P and fruit yield in *C. maxima* plants. These results suggest that a delicately balanced nutrient system is critical for the host to encounter HLB-pathogen invasion.

In conclusion, to our knowledge, this study provides the first estimate of the nutrient resorption process under the specific circumstance, i.e. the HLB-bacterial infection. The findings in the study can help us to better understand the responses of different citrus species to HLB. Different citrus species exhibited the different abilities to tolerate the HLB-pathogen infection. Therefore, it is quite necessary for HLB research to focus on different citrus species. In our study, the citrus species displayed plastic responses to the infection in terms of nutrient resorption. The molecular mechanisms of the resorption process driven by HLB-pathogen infection deserve a further study.

## Methods

### Study sites

This study was conducted in three groves growing with mandarin (*Citrus reticulata* Blanco cv. Shatangju), Eureka lemon (*Citrus limon* (L.) Burm. f.) and pamelo (*Citrus maxima* (Burm.) Merr. cv. Shatian Yu), respectively. The locations and general information of the groves are presented in Table 4. These citrus plants are the evergreen species that flush in early spring, summer and autumn, and produce ripened fruits in autumn.

### Sampling and laboratory analyses

In June 2013, the time of summer shoots immerging in the study areas, we visually identified five ‘*Ca*. L. asiaticus’-infected trees according to leaf symptoms and nearby five healthy trees as controls in each of the three groves. With the help of a skilled colleague, we carefully selected the healthy and HLB-pathogen infected trees following the method by Trivedi *et al.*^43^ to make sure that all the infected trees were in the similar stages of infection. The infected and healthy trees were further confirmed by leaf ‘*Ca*. L. asiaticus’ detection using PCR analysis.

For leaf nutrient analysis, 30 fully expanded live leaves (full sun) and 30 freshly senesced leaves were collected randomly from the canopy of each tree and were separately packed into a brown-paper envelope. Soil samples (0–10 cm depth) were also collected under each tree, 30–50 cm away from the trunk. An auger of 3 cm in diameter was used to collect three cores per tree. The three cores were mixed into a single composite sample *in situ* for each tree, and packed into sealed plastic bags. All samples were kept in a cooler until transported to the laboratory.

In October 2013, the time when autumn shoots grew, we revisited the same trees that were sampled in June 2013, and collected leaf and soil samples for the same processing and analyzing as the summer samples (including PCR confirmation of ‘*Ca*. L. asiaticus’). At the sampling time, the fruits were ready to be harvested. We counted the number of all fruits on the sampling tree, and randomly picked 30 fruits (10 for *C. maxima*) and weighed them, in order to estimate the average fruit yield per tree (i.e. the average weight per fruit multiplies by the total number of fruits on each sampling tree). The fruit yield of *C. limon* trees were not recorded because of the harvest prior to the sampling.

The PCR analysis further confirmed that all the sampled healthy-trees were ‘*Ca*. L. asiaticus’-negative and all the sampled infected-trees were ‘*Ca*. L. asiaticus’-positive both in the summer and in the autumn.

### Chemical analyses of leaf nutrients and soil property

All samples were transported to the laboratory at the day of collecting. The leaf samples were carefully cleaned using deionized water, dried at 65 °C for 48 h, and finely ground with a ball mill (NM200, Retsch, Haan, Germany). Total N in leaf was measured by dry combustion on a CN analyzer (vario El III, Elementar Analysensysteme GmbH, Germany). Total foliar P was analyzed using a concentrated sulfuric acid/hydrogen peroxide digest and an ascorbic acid molybdate colorimetric analysis on a Flow Injection Analysis (AA3, SEAL Analytical GmbH, Germany)^44^.

Field-moist soil samples were sieved through a 2 mm mesh size in order to remove plant materials, small insects and stones. A subsample of field-moist soil was stored in 4 °C refrigerator for determining soil water content, pH and available nutrients within a week. Soil water content was determined by drying for 24 h at 105 °C. Soil pH was determined in a 1:2 soil-to-water (w/v) ratio in deionized water. Available N (ammonium and nitrate) was extracted with 2 M KCl and measured by the AA3^45^. Available P (Olsen’s P) was extracted with 0.5 M NaHCO_3_ (pH = 8.5) and analyzed on the AA3. The rest of soil samples were air-dried and ground to pass through a 100-mesh sieve. Soil organic C and total N concentrations were determined using the method for leaf N.

Leaf and soil nutrient concentrations were expressed on a dry mass basis.

### Nutrient resorption calculations

Nutrient resorption is examined as resorption efficiency and resorption proficiency, following the descriptions from Killingbeck^29^ and Kobe *et al.*^46^. Resorption efficiency is calculated using the two equations below^46^.


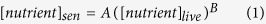



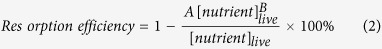


where *A* and *B* are parameters estimated from the measured [*nutrient*]_*live*_ and [*nutrient*]_*sen*_ by regressions, *B* ≠ 1, and [*nutrient*]_*live*_ and [*nutrient*]_*sen*_ represent nutrient concentrations in live leaves and senesced leaves, respectively^46^. Resorption proficiency is the level to which nutrients have been reduced in senesced leaves and is quantified using the senesced-leaf nutrient concentrations. Lower senesced-leaf nutrient concentration indicates higher nutrient resorption proficiency^29^.

Live leaves and senesced leaves were sampled with the consideration of similarity in order to avoid biasing caused by the possible reduction in mass and size during leaf senescence^47^,^48^. We estimated leaf weight by drying and leaf area using portable area meter (LI-3000A), and found that there was no significant difference in specific leaf area between live leaves and senesced leaves in healthy and ‘*Ca*. L. asiaticus’-infected citrus species. Thus, we analyzed nutrient resorption on a mass basis.

### Statistical analysis

All data were tested for normality and homoscedasticity; if either assumption was violated, data were log transformed before analysis. We performed repeated measures analyses of variance based on mixed models to detect significant differences in each response variable through citrus health status, species and sampling dates. The significant differences in variables between ‘*Ca*. L. asiaticus’-infected and healthy citrus plants were analyzed using paired *t*-tests. Means were separated by Duncan’s multiple comparison among the species. Pearson correlation was performed to examine the relationships between the variables measured. All statistical analyses were performed using SAS system for Windows V9.0 (SAS Institute, Cary, North Carolina, USA).

## Additional Information

**How to cite this article**: Cao, J. *et al.* Pathogen infection drives patterns of nutrient resorption in citrus plants. *Sci. Rep.* **5**, 14675; doi: 10.1038/srep14675 (2015).

## Figures and Tables

**Table 1:** Leaf N and P levels in healthy and ‘*Candidatus* Liberibacter asiaticus’-infected citrus plants.

**Species**	**N**		**P**	
	**Healthy**	**Infected**	**Healthy**	**Infected**
**Jun.**				
* C. reticulata*	Optimum	Deficient	Optimum	Low
* C. limon*	Deficient	Low	High	High
* C. maxima*	Optimum	Optimum	High	High
**Oct.**				
* C. reticulata*	Optimum	Optimum	Optimum	High
* C. limon*	Deficient	Low	High	High
* C. maxima*	Optimum	Optimum	High	High
Criteria*				
* *Deficient (%)	<2.2		<0.09	
* *Low (%)	2.2–2.4		0.09–0.11	
* *Optimum (%)	2.5–2.7		0.12–0.16	
* *High (%)	2.8–3.0		0.17–0.30	
* *Excess (%)	>3.0		>0.30	

^*^From Thomas A. Obreza and Kelly T. Morgan, 2011. Nutrition of Florida Citrus Trees, 2nd. http://edis.ifas.ufl.edu/pdffiles/SS/SS47800.pdf.

**Table 2:** *P* values of repeated measures ANOVA for leaf nutrient variables and resorption parameters as dependent on species identity, health status and their interactions.

	**Nliv**	**Nsen**	**NRE**	**Pliv**	**Psen**	**PRE**	**N:Pliv**	**N:Psen**
Species	<0.0001	<0.0001	<0.0001	<0.0001	<0.0001	<0.0001	<0.0001	<0.0001
Status	<0.0001	<0.0001	<0.0001	0.0141	<0.0001	0.001	0.0259	0.9185
Species*Status	<0.0001	<0.0001	<0.0001	<0.0001	<0.0001	<0.0001	<0.0001	0.7334
Date	<0.0001	<0.0001	<0.0001	<0.0001	<0.0001	<0.0001	<0.0001	0.003
Species*Date	<0.0001	<0.0001	<0.0001	<0.0001	<0.0001	<0.0001	<0.0001	<0.0001
Status*Date	<0.0001	0.5720	<0.0001	<0.0001	0.0623	<0.0001	0.0020	0.0999
Species*Status*Date	<0.0001	0.0591	<0.0001	<0.0001	0.1727	<0.0001	<0.0001	0.6414

Nliv and Pliv are N and P concentrations in live leaves, respectively; Nsen and Psen are N and P concentrations in senesced leaves, respectively; NRE and PRE represent N and P resorption efficiencies, respectively; N:Pliv and N:Psen represent N:P ratios in live and senesced leaves, respectively.

**Table 3:** Pearson correlation coefficients of the measured variables (*, ** and *** denote the significant levels at *P* < 0.05, 0.01 and 0.001, respectively).

	**Nsen**		**NRE**		**Pliv**		**Psen**		**PRE**		**N:Pliv**		**N:Psen**	
	**Healthy**	**Infected**	**Healthy**	**Infected**	**Healthy**	**Infected**	**Healthy**	**Infected**	**Healthy**	**Infected**	**Healthy**	**Infected**	**Healthy**	**Infected**
Nliv	−0.744***	−0.528**	0.955***	0.930***	−0.314	0.659***	−0.880***	−0.407*	0.405*	0.845***	0.660***	−0.352	0.674***	0.058
Nsen			−0.895***	−0.758***	0.269	−0.265	0.748***	0.440*	−0.374*	−0.462*	−0.515**	0.105	−0.253	0.263
NRE					−0.339	0.634***	−0.894***	−0.443*	0.404*	0.832***	0.658***	−0.368*	0.555**	−0.075
Pliv							0.21	0.128	0.667***	0.898***	−0.913***	−0.931***	−0.024	−0.305
Psen									−0.572***	−0.278	−0.528**	−0.344	−0.817***	−0.738***
PRE											−0.361*	−0.730***	0.556**	−0.014
N:Pliv													0.295	0.414*

Nliv and Pliv are N and P concentrations in live leaves, respectively; Nsen and Psen are N and P concentrations in senesced leaves, respectively; NRE and PRE represent N and P resorption efficiencies, respectively; N:Pliv and N:Psen represent N:P ratios in live and senesced leaves, respectively. The pairs of variables with positive correlation coefficients and *P* values below 0.050 tend to increase together. For the pairs with negative correlation coefficients and *P* values below 0.050, one variable tends to decrease while the other increases. For pairs with *P* values greater than 0.050, there is no significant relationship between the two variables.

**Table 4:** Description of the three sampling sites of this study in Guangdong, China.

**Site**	**Species**	**Latitude**	**Longitude**	**Altitude (m)**	**MAT (°C)**	**MAP (mm)**	**pH**	**SOC (mg g**^**−1**^)	**TN (mg g**^**−1**^)	**AN (mg kg**^**−1**^)	**AP (mg kg**^**−1**^)
Huizhou	*Citrus reticulata*	23^o^16.613′N	114^o^15.737′E	63	22.3	1897.0	4.62 (0.14)	10.17 (1.40)	0.930 (0.13)	23.82 (4.90)	18.73 (3.40)
Heyuan	*Citrus limon*	24^o^05.947′N	114^o^45.396′E	141	21.0	1742.0	4.38 (0.09)	11.49 (2.09)	0.927 (0.07)	28.77 (4.98)	4.27 (1.07)
Meizhou	*Citrus maxima*	24^o^26.070′N	116^o^05.853′E	178	21.3	1528.6	4.76 (0.57)	9.92 (2.35)	1.272 (0.22)	17.40 (2.89)	56.58 (6.13)

Values are means with standard errors in parentheses, n = 5. MAT, mean annual temperature; MAP, mean annual precipitation; SOC, soil organic carbon; TN, total nitrogen; AN, available nitrogen; AP, available phosphorus.
